# Filtered, yet not homogenised: Urbanisation does not lead to cross‐city convergence in Mexican urban bird communities

**DOI:** 10.1111/1365-2656.70311

**Published:** 2026-09-01

**Authors:** Ian MacGregor‐Fors, Christine C. Rega‐Brodsky, Bruno Flório Lessi, Jorge E. Schondube

**Affiliations:** ^1^ Department of Environmental Sciences, Faculty of Biological and Environmental Sciences University of Helsinki Helsinki Finland; ^2^ School of Natural Resources, University of Missouri Columbia Missouri USA; ^3^ Neotropical Birds Lab, Department of Ecology and Evolutive Biology Federal University of São Carlos—UFSCar São Carlos Brazil; ^4^ Instituto de Investigaciones en Ecosistemas y Sustentabilidad, Universidad Nacional Autónoma de México Morelia Michoacán México

**Keywords:** beta diversity, biotic homogenisation, composition, filtering, functional, species richness, taxonomic, urban ecology

## Abstract

1. Urbanisation acts as a non‐random filter of biodiversity, often resulting in less diverse communities dominated by a subset of species with particular traits. These patterns have led to the expectation that urban environments may promote biotic homogenisation, yielding taxonomically or functionally similar communities across cities, relative to their surrounding natural ecosystems.
2. Here, we evaluated whether the non‐random filtering of biodiversity by urbanisation results in the homogenisation of bird communities across three environmental conditions (i.e. natural ecosystems, urban greenspaces, urban sites) in three biogeographically distinct Mexican cities.
3. Assuming an urban‐driven filtering process (i.e. an overall decline in native species richness from natural ecosystems into the urban matrix, with possible additions of exotic species), we formulated scenario‐based expectations to disentangle urban filtering from biotic homogenisation, predicting whether filtering results in locally distinct urban communities, or additionally, convergent filtering across cities drives cross‐city homogenisation.
4. We quantified taxonomic and functional richness (alpha diversity) and compositional dissimilarity (beta diversity) across natural ecosystems, urban greenspaces and urban sites to evaluate whether only filtering occurred or if such process has additionally led to context‐dependent convergence in community composition across cities, which could indicate homogenisation. We analysed taxonomic and functional approaches using incidence‐ and abundance‐based metrics to assess compositional variation within and among cities.
5. We recorded 131 bird species, of which only a handful were shared across urban conditions in all three cities. Bird communities showed strong taxonomic and functional differentiation among cities, with greater similarity within the studied environmental conditions per city region than across cities, indicating that urbanisation filters species locally, with no clear evidence of cross‐city convergence in community composition.
6. Our findings indicate that urbanisation filters species locally, simplifying communities, yet avian assemblages remain more similar within the three environmental conditions per city region than across the three surveyed cities in Mexico. Notably, widespread and abundant urban‐associated species contribute to increased similarity within urban assemblages but do not result in cross‐city convergence. This challenges the notion that urbanisation causes homogenisation (at least across the three studied Mexican cities and their surrounding natural ecosystems, which belong to highly distinct biogeographic regions) and highlights the predominant influence of regional species pools and local environmental contexts in shaping urban avifaunas.

## Graphical abstract

Urbanisation simplifies bird communities, but does not necessarily make urban avifaunas alike. Across three biogeographically distinct Mexican cities, urban bird assemblages remained regionally distinct despite strong local filtering. These results challenge assumptions of urban‐driven homogenisation and emphasise the dominant role of regional species pools and local environmental context.

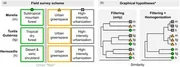

## 1 INTRODUCTION

Three decades of urban ecological research reveal that urbanisation and the metabolism of cities (i.e. the flow of energy, materials and resources that sustain urban activities; Kennedy et al., 2011) exert immense pressure on the biodiversity capable of persisting within urban environments (Czech & Krausman, 1997; Maxwell et al., 2016; Rega‐Brodsky et al., 2022). It has been widely documented that urbanisation acts as a non‐random ecological filter, resulting in communities with lower species richness than those non‐urban counterparts, often dominated in abundance by a small number of species (Aronson et al., 2014; Croci et al., 2008). This non‐random reduction of wildlife communities in urban areas is generally attributed to species‐specific traits, often linked to ecological function and phylogenetic history, which mediate tolerance to urban conditions and shape population persistence outcomes (La Sorte et al., 2018; MacGregor‐Fors et al., 2022; Sol et al., 2014). Such adaptive differences among species, rather than random processes, primarily drive patterns of species loss (Aronson et al., 2016; Evans et al., 2011; Lapiedra et al., 2013; Silva et al., 2016).

One emergent pattern that has been proposed as a consequence of this non‐random filtering process is biotic homogenisation. Since McKinney's (2006) seminal work, urbanisation has been widely and often implicitly linked to biotic homogenisation, defined as the ‘process of replacing localized native species with increasingly widespread non‐native species’. McKinney argued that by altering urban physical environments, cities would favour the persistence and shared presence of urban‐adapted species across different urban areas (McKinney, 2006). Over time, however, the definition of biotic homogenisation has evolved. Early descriptions emphasised the replacement of local endemic species and regionally distinct communities with non‐local species and cosmopolitan communities (Clergeau et al., 2006; McKinney & Lockwood, 1999). Later studies shifted focus to assessing the increasing taxonomic similarity among sites, as evidenced by a temporal decline in beta diversity (Olden & Rooney, 2006). These evolving definitions and varied approaches to biotic homogenisation have introduced challenges in interpreting studies on the topic over time (Yang et al., 2026).

The conceptualisation of biotic homogenisation in urban systems has triggered a wave of research over the past two decades, generally framed in terms of compositional similarity among communities. Notably, although some studies refer to it in different ways, beta diversity comprises multiple facets (i.e. differentiation diversity, turnover, proportional diversity‐scaling; Moreno & Rodríguez, 2010), each measured differently and requiring distinct interpretation. Following this conceptual framework, researchers have utilised some of these facets of beta diversity to test for the presence of biotic homogenisation associated with urbanisation in their study sites (Le Tortorec et al., 2023; Lokatis & Jeschke, 2022), often using similarity or dissimilarity indices as measures of differentiation and turnover (Catterall et al., 2010; Hensley et al., 2019; Leveau et al., 2017; Morelli et al., 2017). Studies exploring urbanisation gradients often report biodiversity loss alongside an increase in generalist species, a trend interpreted as both biotic homogenisation and as a further manifestation of spatio‐temporal filtering driven by urbanisation (Concepción et al., 2015; Hensley et al., 2019; Silva et al., 2016; Sol et al., 2014). Notably, much of this research focuses on local‐to‐regional scales (Evans et al., 2018; Le Tortorec et al., 2023; Leveau et al., 2015; Leveau & Leveau, 2012; Lizée et al., 2011).

Efforts to understand and categorise species' responses to urbanisation have long focused on birds, often used as bioindicators (Lokatis & Jeschke, 2022; Rega‐Brodsky et al., 2022). Beyond local‐to‐regional scale studies claiming to assess or demonstrate biotic homogenisation driven by urbanisation, few studies have used global datasets to investigate avian biotic homogenisation, for which the following two are illustrative. Aronson et al. (2014), using an incidence‐based dataset, found that while some exotic species are widely shared among cities globally, urban birds have not yet become fully taxonomically homogenised and continue to reflect their regional species pools. Conversely, Sol et al. (2014), analysing abundance changes between urbanised environments and their non‐urbanised surroundings at a global scale, showed that although some species loss associated with urbanisation may occur randomly, much of it is driven by species' biological traits and their ability to exploit urban resources and avoid urban‐associated risks. They further concluded that, by filtering species according to these traits, urban environments can reduce beta diversity and thereby promote biotic homogenisation (Sol et al., 2014). These findings, which may appear contrasting, underscore the mixed support for the biotic homogenisation hypothesis and highlight the need for further research to resolve these discrepancies (Lokatis & Jeschke, 2022).

In this study, we aimed to determine whether urbanisation acts as an ecological filter, reducing native species richness and functional diversity, and whether this filtering process ultimately drives biotic homogenisation of bird communities across three ecologically distinct Mexican cities, making urban assemblages more similar to each other than to their regional natural ecosystems, even under ecologically distinct scenarios. Although introductions of exotic species represent a distinct process that could also contribute to homogenisation (Aronson et al., 2014; Sol et al., 2014), we treated them as part of the urban bird assemblage as a whole. Because biotic homogenisation, by definition, involves a temporal component (McKinney, 2006; Olden & Rooney, 2006), and given the lack of time‐series data to directly assess temporal change, we employed a space‐for‐time substitution approach (Pickett, 1989). Specifically, we surveyed remnant natural ecosystems assumed to represent pre‐urban conditions and compared them with their contemporary urbanised counterparts. Thus, we assessed the expected decay in species richness and the similarity of bird communities, both taxonomically and functionally, across urban sites, greenspaces and natural ecosystems in three biogeographically distinct regions of Mexico.

Examining functional alongside taxonomic diversity is particularly important because changes in functional composition may indicate a simplification of ecological roles, such as the replacement of ecological specialists by widespread generalists, which might occur even in the absence of strong taxonomic homogenisation (Morelli et al., 2016; Olden et al., 2004). Therefore, analysing both taxonomic and functional patterns allowed us to capture not only species loss, but also shifts in ecological roles that may be driven by urbanisation. Our study sites (i.e. Hermosillo: arid, northern Mexico; Morelia: subtropical mountain, central Mexico; Tuxtla Gutiérrez: lowland tropical, southern Mexico) were selected to maximise variability in species pools, allowing a robust test of whether urban avifaunas are filtered, maintaining the distinctiveness of their regional assemblages, or whether filtering has also led to biotic homogenisation, making urban communities more similar to each other than to their respective natural ecosystems.

We formulated the following scenario‐based hypotheses (Figure 1). If biotic homogenisation is occurring across our study sites, urban bird communities, both taxonomically and functionally, would be more similar to each other across cities than to their regional natural ecosystems, reflecting not only shared species identities but also a convergence in functional roles. Conversely, if species richness declines with urbanisation while urban communities remain both taxonomically and functionally distinct between cities, this pattern would indicate that filtering has not yet resulted in homogenisation, representing filtering without convergence (Aronson et al., 2016; MacGregor‐Fors et al., 2022). Given that the most abundant urban bird species in Mexico can dominate total counts (MacGregor‐Fors et al., 2012), we considered both incidence and abundance‐weighted procedures for both taxonomic and functional approaches. Thus, if results are consistent across these measures, conclusions are straightforward. However, if abundance‐based measures show higher similarity than incidence‐based ones, this likely reflects the dominance of a few highly urban‐associated species, which can drive functional convergence even if taxonomic similarity is limited, while many other, less abundant species are underrepresented in abundance‐weighted metrics. In line with Cassey et al. (2008), such patterns are typical of communities strongly perturbed by urbanisation or other disturbances, where the dominance of a few widespread species can disproportionately drive similarity, causing abundance‐based metrics to suggest homogenisation even when presence–absence data indicate otherwise. These patterns would therefore not necessarily reflect consistent biotic homogenisation across the entire community, but instead indicate abundance‐driven similarity. In such cases, similarity is mostly shaped by the most abundant species, while moderately abundant and rare species maintain regional distinctiveness in both taxonomic identities and functional roles.

**FIGURE 1:**
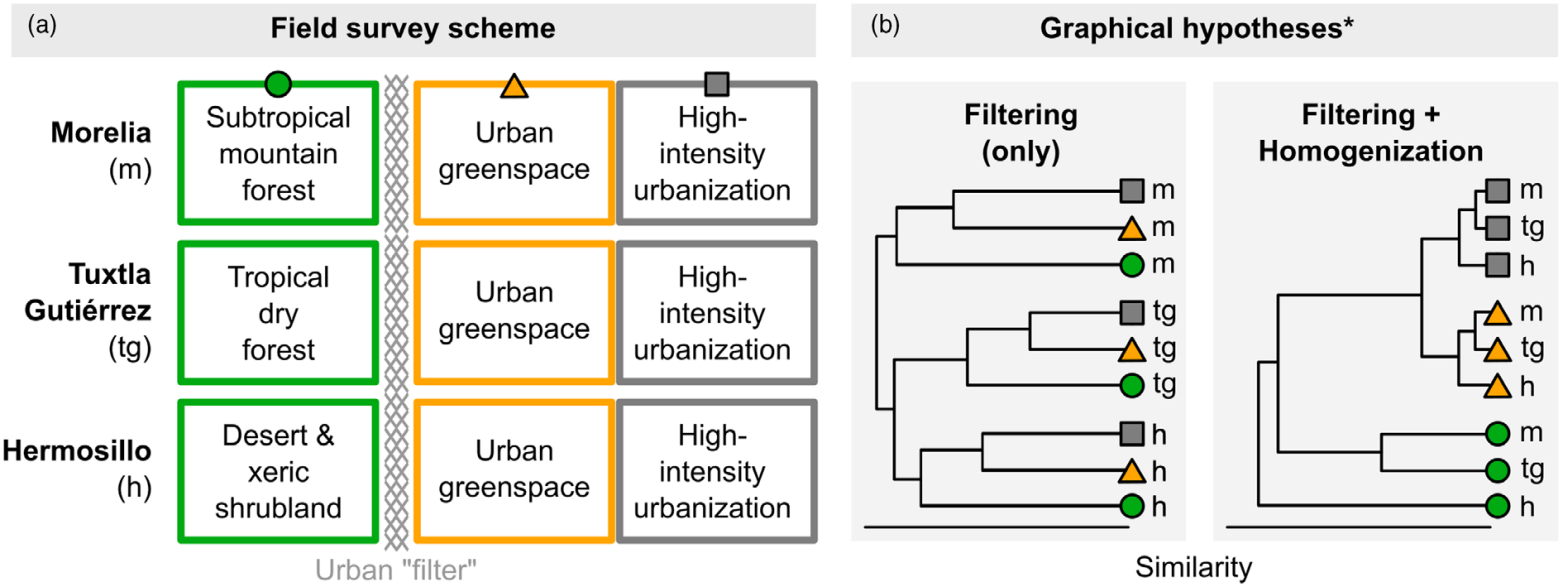
(a) Conceptual representation of the field survey scheme and expected compositional patterns along the studied environmental conditions, illustrating the assumed filtering of species pools from natural ecosystems into urban greenspaces and urban sites across three studied Mexican cities. Based on the assumed filtering (expected decay in species richness between natural ecosystems and sites within the urban matrix), we present two graphical hypotheses (*) of community composition to provide an explanatory framework (b): (i) urban filtering only, leading to low similarity among urban communities across cities and taxonomic and functional clustering within city regions; and (ii) urban filtering combined with biotic homogenisation, resulting in high similarity among urban communities, particularly urban sites, across city regions.

## 2 MATERIALS AND METHODS

### 2.1 Study areas

We conducted this study in three medium‐sized Mexican cities within contrastingly different landscapes: Hermosillo, Morelia and Tuxtla Gutiérrez (Figure 2). These cities are located in the northernmost, centre and southernmost regions of Mexico, respectively, representing distinct biogeographic regions. Hermosillo, the state capital of Sonora, is situated in northwestern Mexico and spans approximately 165 km^2^. The city has experienced rapid urban expansion, marked by significant population growth (Ortega‐Rosas et al., 2020), with an estimated population of 936,000 inhabitants (INEGI, 2020). The region is characterised by a hot desert climate, with high temperatures often exceeding 40°C in summer and limited rainfall. Surrounded by arid landscapes, Hermosillo's natural ecosystems include deserts and xeric shrublands, offering a stark contrast to its urbanised areas.

**FIGURE 2:**
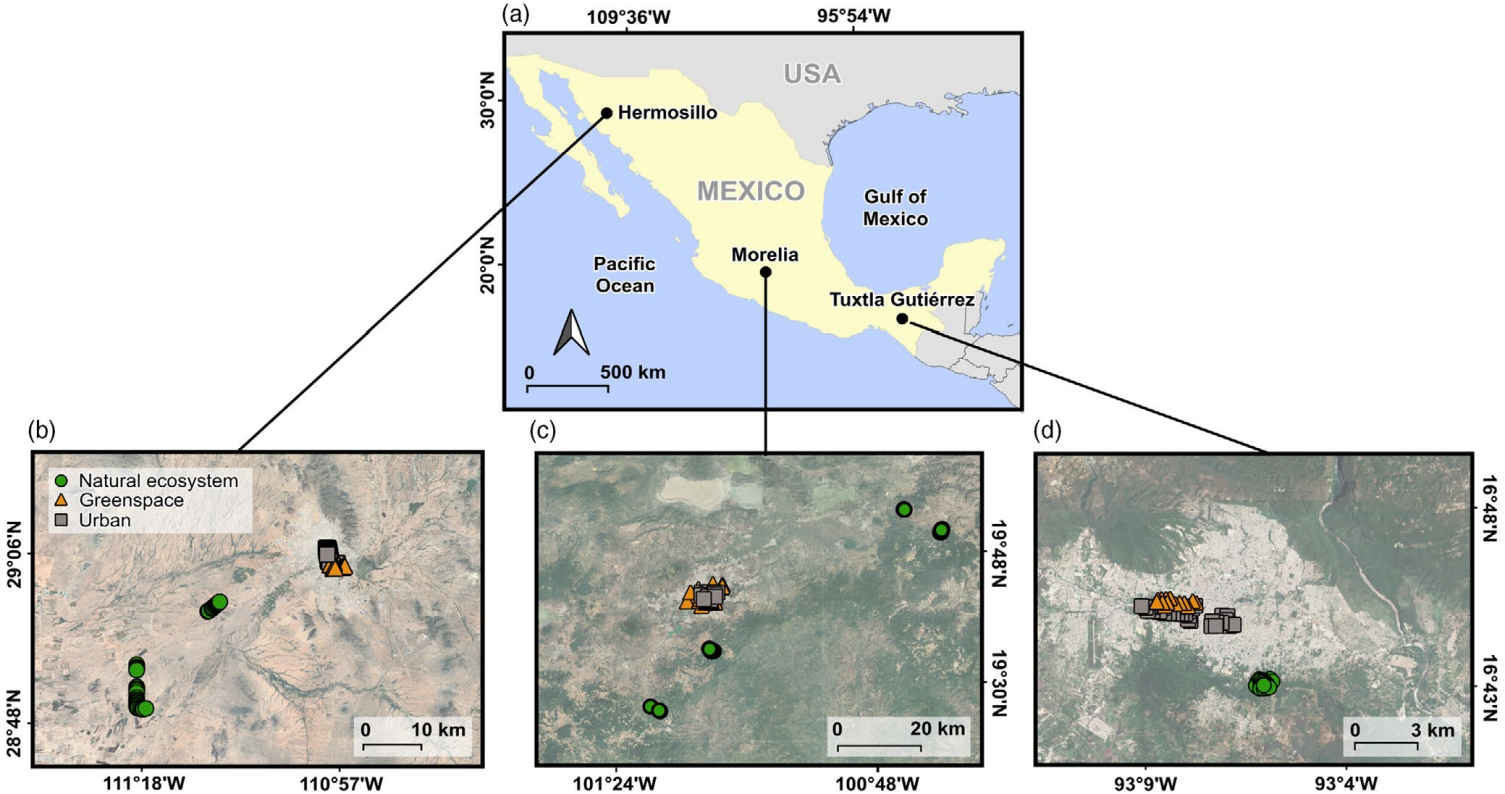
(a) Location of the studied Mexican cities and surrounding natural ecosystems: (b) Hermosillo, (c) Morelia, and (d) Tuxtla Gutiérrez.

Morelia, state capital of Michoacán, covers an area of approximately 130 km^2^ and has a population of 849,000 inhabitants (INEGI, 2020). The city is predominantly made up of residential and commercial areas, with few large greenspaces contributing to the overall vegetated land cover (López et al., 2001). Morelia experiences a subtropical highland climate, characterised by mild temperatures and a distinct wet and dry season. The surrounding natural ecosystems are dominated by subtropical mountain forests.

Tuxtla Gutiérrez, the capital and largest city of Chiapas, spans approximately 90 km^2^ and has a population of 580,000 residents (INEGI, 2020). Tuxtla Gutiérrez experiences a tropical climate with distinct wet and dry seasons, with high temperatures year‐round. The city is surrounded by Natural Protected and Conservation Areas, which are predominantly characterised by tropical dry and semideciduous forests, offering a rich diversity of tropical species (Castro‐Mendoza et al., 2022; ICIPLAM, 2013).

### 2.2 Sampling design and data collection

To test whether urban bird communities are only filtered, or additionally homogenised by urbanisation, considering both heavily urbanised and well‐vegetated areas within cities, we surveyed three environmental conditions across each city's region: high‐intensity urbanisation, low‐intensity urbanisation and natural ecosystems. High‐intensity urbanisation (hereafter referred to as ‘urban’) was represented by residential and commercial areas with minimal vegetation cover (>70% built cover). Low‐intensity urbanisation (hereafter referred to as ‘greenspaces’) included urban parks and other greenspaces with more than 50% vegetation cover. Both urban and greenspace locations were situated within the intra‐urban areas of the cities to reduce potential peri‐urban influences (sensu MacGregor‐Fors, 2010). Natural ecosystems, used as city‐region controls, consisted of areas located 10–50 km from city centres in Morelia and Hermosillo, characterised by well‐preserved native vegetation typical of each region. Due to security and mobility constraints, the natural ecosystem in Tuxtla Gutiérrez was restricted to the protected natural reserve El Zapotal, which retains important regional biodiversity, serving as a representative reference of regional avifauna. Surveys were conducted in El Zapotal with authorised access and accompanied by a local guide.

An experienced field ornithologist with expert knowledge of the avifauna of the regions studied (IM‐F) surveyed breeding birds during June and July 2008 using the point‐count technique (5 min, unlimited radius; 1 visit per site), conducted from sunrise to 4 h after (Ralph et al., 1996). All birds seen or heard within the surveyed area were recorded, excluding flyovers. Point‐count locations were at least 250 m apart to minimise the likelihood of spatial dependence (Bibby et al., 2000). Survey locations were chosen based on the feasibility of accessing public spaces, while considering security concerns. One point‐count survey was conducted at 30 sites within each city's respective environmental condition (i.e. urban, greenspaces and natural ecosystems), for a total of 270 surveyed sites.

### 2.3 Data analysis

To quantify variation in species richness, we conducted a rarefaction analysis to estimate cumulative bird species richness in all studied environmental conditions per city region (i.e. urban, greenspaces and natural ecosystems). This analysis was performed with 1000 bootstrap replications using the iNEXT R package (Hsieh et al., 2016), following the procedures of Gotelli and Colwell (2001). We assessed statistical differences between conditions across each city's region by evaluating whether 84% confidence intervals (CIs) overlapped. This approach provides an approximate two‐tailed test at *α* = 0.05, based on the property that non‐overlapping 84% CIs correspond closely to a conventional significance test at the 5% level, including asymmetric distributions (MacGregor‐Fors & Payton, 2013). To quantify functional richness, we followed the framework of Laliberté et al. (2014), which defines it as the volume of multidimensional trait space occupied by a community. We characterised ecological function using three traits: dominant diet, body size (g) and foraging stratum, with trait information obtained from Wilman et al. (2014). For foraging stratum, we assigned numeric values reflecting vertical foraging position (1 = ground, 2 = understory, 3 = mid‐high, 4 = canopy and 5 = aerial) and calculated a weighted value from the percentages in the dataset, producing a single numerical value for each species, ranging from 1 to 5, where 1 corresponds to exclusively ground foraging and 5 to exclusively aerial foraging. Trait values were combined with the abundance matrix to compute a Gower distance matrix, and a principal coordinates analysis (PCoA) was performed to obtain orthogonal trait axes and calculate the proportion of variance explained by each axis. We selected the minimum number of axes needed to retain at least 80% of the cumulative variance, and this reduced trait space was used to estimate functional species richness for each community following Laliberté and Legendre (2010) and Laliberté et al. (2014). Functional richness was calculated separately for each studied condition (i.e. urban, greenspaces and natural ecosystems). For comparability, functional richness values were then normalised by dividing each community's value by the maximum value observed across all conditions.

To evaluate taxonomic and functional composition across study sites, we used incidence‐ and abundance‐based approaches, depending on the data type and analytical framework. For taxonomic composition, incidence‐based dissimilarity was computed using the Sørensen index (*β*_sor_; Baselga & Leprieur, 2015), while abundance‐based dissimilarity was calculated with the Bray–Curtis index (Bray & Curtis, 1957), using the full set of surveyed sites (*n* = 270). Functional composition was assessed using the same species traits as those used for functional richness estimation, projected into a reduced‐dimensionality trait space obtained from a PCoA of a Gower distance matrix. For incidence‐based functional analyses, we used the functional Sørensen index (functional *β*_sor_; Laliberté & Legendre, 2010), whereas for abundance‐based functional analyses, functional dissimilarity in community‐weighted mean (CWM) trait space was calculated using Euclidean distances. Because the calculation of functional beta diversity requires that each site contains more data (species or abundances) than retained functional axes, sites with species richness equal to or lower than the number of retained functional axes were excluded. Consequently, functional assessments were conducted using a subset of sites for which these calculations were feasible (incidence‐based *n* = 212 sites; abundance‐based *n* = 258 sites).

To statistically test differences in taxonomic and functional composition among study categories (i.e. urban, greenspaces and natural ecosystems) and across city regions, we performed permutational multivariate analyses of variance (PERMANOVA; ‘adonis2’ function, R package ‘vegan’, 999 permutations; Oksanen et al., 2012). Pairwise comparisons between categories were conducted with a Benjamini–Hochberg correction for multiple testing, which controls the false discovery rate, while maintaining statistical power across multiple pairwise contrasts (Benjamini & Hochberg, 1995). Additionally, we calculated average pairwise dissimilarities between categories and used hierarchical clustering (average linkage) to visualise patterns of similarity among site categories for both taxonomic and functional datasets. This approach allowed us to assess whether urban sites differ in compositional structure relative to their respective natural ecosystems, both taxonomically and functionally (as depicted in the graphical hypotheses of Figure 1).

## 3 RESULTS

We recorded a total of 131 bird species across the three studied city regions (Table S1). Considering all study enironmental conditions together (i.e. urban, greenspaces and natural ecosystems), Morelia had the greatest avian species richness (63 species), followed by Tuxtla Gutiérrez (52 species) and finally Hermosillo (46 species; Table 1). In all cases, we recorded significantly higher taxonomic bird richness in natural ecosystems compared to greenspaces and urban categories (Figure 3a). In Morelia and Tuxtla Gutiérrez, greenspaces also had significantly higher species richness than urban categories. However, in Hermosillo, no significant differences in species richness were observed between the greenspaces and urban categories (Figure 3a). Regarding functional richness, natural ecosystems consistently exhibited higher values than both urban categories (i.e. greenspaces and urban sites). However, while functional richness declined gradually from natural ecosystems to greenspaces and urban sites in Morelia and Tuxtla Gutiérrez, urban sites in Hermosillo exhibited higher functional richness than Hermosillo's greenspaces (Figure 3b), indicating context‐dependent variation in functional filtering across cities. Notably, taxonomic and functional richness were tightly correlated (Pearson *r* = 0.87, df = 7, *p* = 0.002).

**TABLE 1:** Total number of recorded bird species in the studied environmental conditions and city regions.

City region	Environmental conditions			City region total
	Natural ecosystem	Greenspace	Urban	
Hermosillo	36	15	19	46
Morelia	42	28	18	63
Tuxtla Gutiérrez	31	26	17	52

**FIGURE 3:**
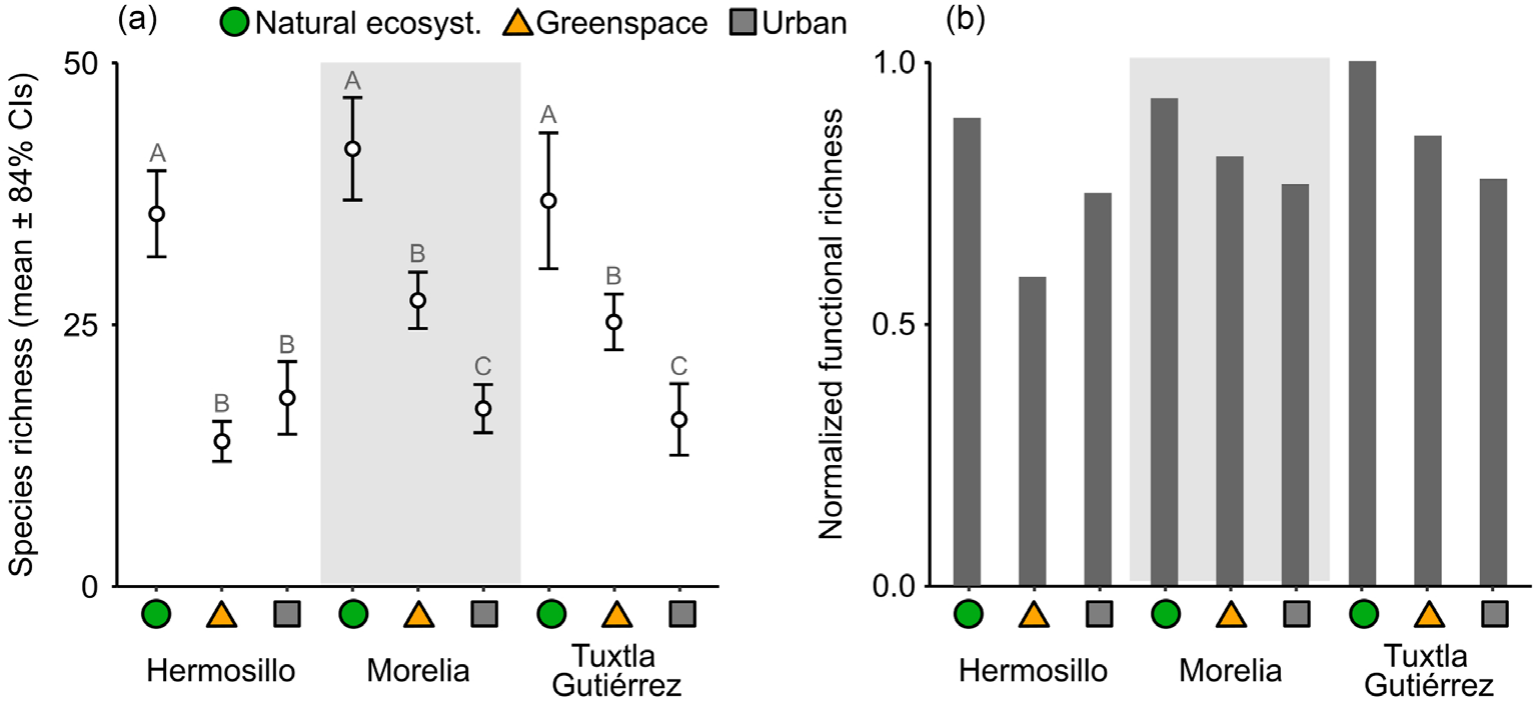
Avian richness contrasts among urban, greenspace and natural ecosystem conditions across the three studied city regions. (a) Taxonomic assessment; differing letters above the confidence intervals (CIs) indicate statistically significant species richness differences within city regions, but not among regions. (b) Functional assessment, depicting normalised functional richness. The shaded area in the comparisons for the Morelia city region is included as a visual aid.

Of the 131 species recorded in this study, only a small subset of widespread, urban‐associated species occurred across cities in both urban sites and greenspaces. House Sparrow (*Passer domesticus*) and Great‐tailed Grackle (*Quiscalus mexicanus*) were the most consistently recorded species across urban sites and greenspaces, although with variation among cities, particularly in Tuxtla Gutiérrez where House Sparrow was rare in greenspaces. Rock Pigeon (*Columba livia*) showed a marked context‐dependent pattern, with higher numbers in greenspaces than urban sites in Hermosillo, but higher numbers in urban sites than greenspaces in Morelia and Tuxtla Gutiérrez. In contrast, Inca Dove (*Columbina inca*) occurred broadly across all studied environmental conditions, but with higher representation and abundance in urban sites and greenspaces.

Composition analyses revealed strong and consistent differences among study conditions across city regions (Figure 4). Incidence‐based taxonomic composition differed significantly among all site categories and cities, with the model explaining nearly half of the variation in Sørensen dissimilarity (PERMANOVA: *F*_8,269_ = 31.45, *r* ^2^ = 0.491, *p* = 0.001). Pairwise comparisons confirmed that all contrasts, both within and between city regions, were statistically significant (*p* < 0.001), consistent with high average dissimilarity values observed for each environmental condition across all city regions (urban sites = 0.757, greenspaces = 0.859, natural ecosystems = 0.989). Abundance‐based taxonomic composition showed a similarly robust pattern (PERMANOVA Bray–Curtis: *F*_8,269_ = 20.50, *r* ^2^ = 0.386, *p* = 0.001), with all pairwise comparisons remaining significant (*p* < 0.001). Despite the lower explained variance, average dissimilarity for each environmental condition across all city regions was higher than in the incidence‐based assessment (urban sites = 0.824, greenspaces = 0.905, natural ecosystems = 0.992).

**FIGURE 4:**
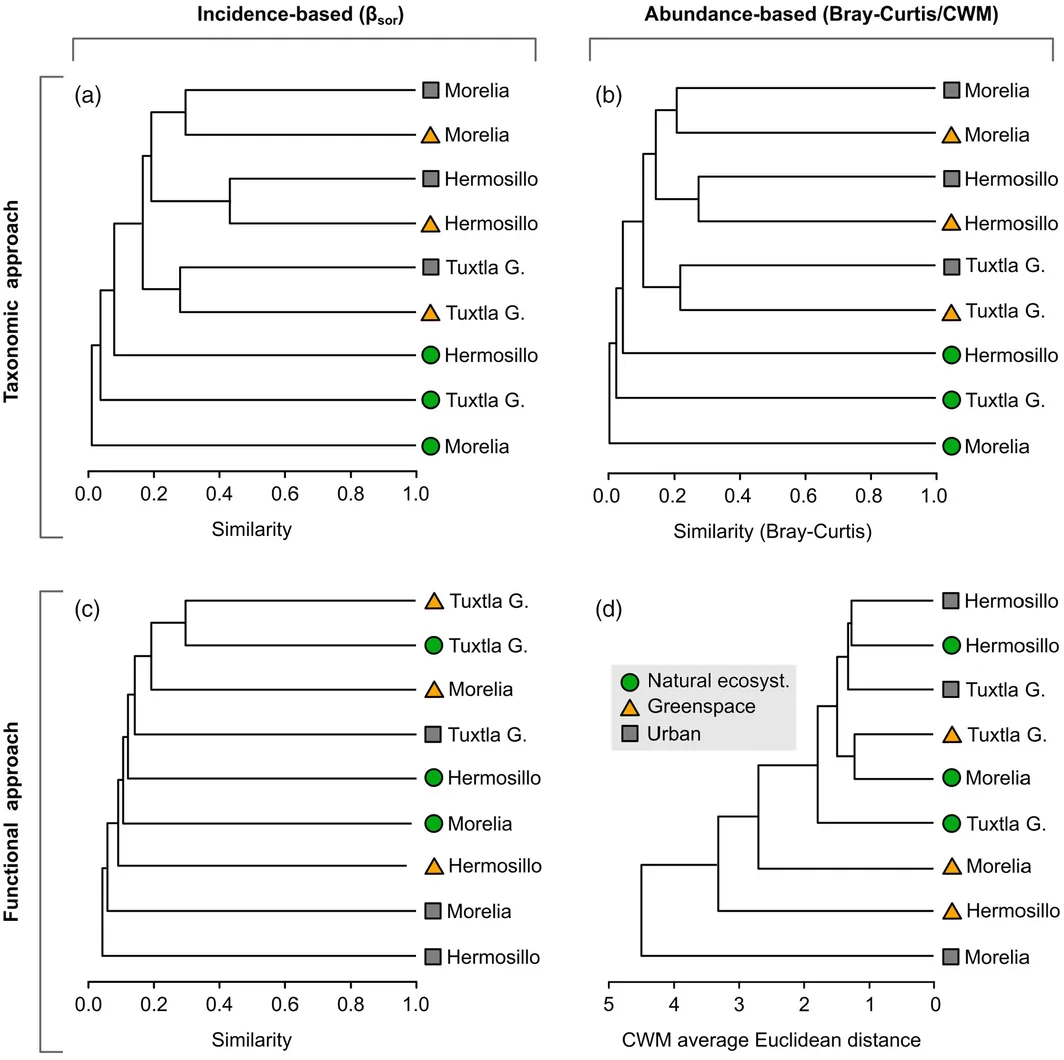
Avian taxonomic (a, b) and functional (c, d) compositional differences, based on incidence (a, c; *β*_sor_) and abundance‐weighted (Bray–Curtis [taxonomic; b] or community‐weighted mean [CWM] functional trait values; d) approaches, across the studied conditions.

Functional composition based on incidence data also differed significantly among study conditions (functional Sørensen PERMANOVA: *F*_8,211_ = 5.95, *r* ^2^ = 0.190, *p* = 0.001), indicating lower explained variance when compared to the taxonomic approach, and weaker and less consistent differentiation among environmental conditions, with some non‐significant pairwise contrasts. Most pairwise functional comparisons were significant (*p* < 0.048) with the exception of several contrasts: Hermosillo natural ecosystems versus Tuxtla Gutiérrez urban sites (*p* = 0.173), Hermosillo greenspaces versus Tuxtla Gutiérrez urban sites (*p* = 0.071), Hermosillo urban versus Morelia urban sites (*p* = 0.080), Morelia natural ecosystems versus Tuxtla Gutiérrez urban sites (*p* = 0.172) and Morelia greenspaces versus Tuxtla Gutiérrez urban sites (*p* = 0.064). Functional composition based on abundance‐weighted traits also differed significantly among study conditions and city regions (PERMANOVA CWM traits: *F*_8,261_ = 5.45, *r* ^2^ = 0.143, *p* = 0.001). Pairwise comparisons indicated that most contrasts were statistically significant (*p* < 0.04), both within and between city regions, although a higher proportion of non‐significant differences was observed compared to the incidence‐based analyses. Non‐significant contrasts were particularly associated with comparisons involving greenspaces and urban sites, including Hermosillo natural ecosystems versus Tuxtla Gutiérrez greenspaces (*p* = 0.055), Hermosillo natural ecosystems versus Tuxtla Gutiérrez urban sites (*p* = 0.206), Hermosillo greenspaces versus Morelia greenspaces (*p* = 0.071), Hermosillo greenspaces versus Morelia urban sites (*p* = 0.552) and greenspaces versus urban sites within Morelia (*p* = 0.396).

Notably, although some pairwise contrasts were not significantly different under both functional composition approaches, these cases highlight the context dependency of the observed patterns, as many involved ecologically distinct systems across city regions. The only non‐significant contrast within the same city region was between greenspaces and urban sites in Morelia, suggesting relatively similar functional composition between these two human‐modified conditions. Nevertheless, these cases should be interpreted within the broader context of generally high functional dissimilarity values across cities and conditions. For incidence‐based functional composition, average dissimilarity values remained high across cities within each condition (urban sites = 0.945, greenspaces = 0.981, natural ecosystems = 0.839). In the case of abundance‐weighted functional composition, average Euclidean distances in CWM trait space across cities within each condition were lowest in natural ecosystems (1.640), but almost twice as high in urban sites (3.156) and greenspaces (3.116).

## 4 DISCUSSION

Hypotheses about biotic and functional homogenisation resulting from urbanisation remain prevalent in the literature, with varying levels of empirical support (Lokatis & Jeschke, 2022). However, recent global analyses suggest that contrary to long‐standing expectations, there is no clear general homogenisation of communities across human‐dominated ecosystems (Keck et al., 2025). Moreover, it has been argued that even the evidence for biotic homogenisation may be influenced by methodological limitations (Yang et al., 2026). To contribute to this discussion, we provide evidence that the studied Mexican urban bird communities are structured by the regional species pool and local environmental filters, in terms of both taxonomic and functional composition, without clear evidence of widespread homogenisation driven by urbanisation. Our results show that bird taxonomic and functional richness are consistently highest in natural ecosystems and decline with increasing urbanisation, while greenspaces partially retain diversity. However, patterns vary among cities, particularly in Hermosillo. Despite these differences in richness, we observed consistent taxonomic and functional compositional dissimilarity across environmental conditions and city regions, with urban and greenspace assemblages dominated by a small set of widespread, urban‐associated species.

Our results reveal a consistent decline in both taxonomic and functional bird richness across the surveyed conditions, from natural ecosystems to urban greenspaces and highly urbanised sites, with natural ecosystems supporting the highest richness and urban sites, generally, the lowest. Greenspaces generally showed intermediate values, indicating that they can partially mitigate richness loss, but do not fully compensate for the effects of habitat transformation, a pattern commonly reported in the literature and likely related to the greater availability of habitat and vegetation relative to highly urbanised areas (Chace & Walsh, 2006; Rega‐Brodsky & MacGregor‐Fors, 2023). However, patterns were not uniform across cities. In Hermosillo, both taxonomic and functional richness were higher in urban sites than in greenspaces, although taxonomic richness did not differ significantly between these categories. This deviation from expected patterns is noteworthy in light of previous work suggesting that rainfall and extreme aridity can act as a macroecological factor shaping bird communities in urban environments (de Albuquerque et al., 2021; Liang et al., 2020; MacGregor‐Fors et al., 2021; Richardson et al., 2023), although cities in arid regions remain comparatively understudied (Diamant et al., 2025). Additionally, we recognise that avian diversity in the Tuxtla Gutiérrez study region would likely have been higher in natural ecosystems; however, continuous, minimally disturbed reference sites were not available. Still, the studied reserve retains important biodiversity and semi‐natural characteristics, but it does not represent such reference conditions. Finally, the strong positive correlation between taxonomic and functional richness suggests that species loss across the surveyed conditions is closely accompanied by reductions in functional trait space, suggesting limited functional redundancy in the studied bird assemblages. Ecologically, this pattern implies that urban‐driven species loss is not buffered by replacement with functionally similar species, but rather translates into a parallel contraction of ecological functions across the urban gradient.

Compositional results indicate that urban bird communities in our study area show no evidence of homogenisation, but instead exhibit high taxonomic and functional turnover shaped by regional species pools and local contexts. Declines in taxonomic and functional richness are in line with urban filtering, as observed globally (Aronson et al., 2014; Sol et al., 2014). Yet, this filtering did not result in homogenisation in our study system. Species overlap across environmental conditions and city regions was limited to a few generalist, urban‐associated species (i.e. Inca Dove, House Sparrow, Great‐tailed Grackle, Rock Pigeon), reflecting overall community patterns rather than generalisable convergence. This interpretation is consistent with the similarity of taxonomic composition results based on incidence and abundance data: abundance‐weighted analyses produced highly similar patterns to those of incidence‐based analyses, indicating that species dominance did not strongly influence compositional differentiation. These findings highlight the importance of methodological choices when assessing biotic homogenisation, as different approaches can yield distinct interpretations (Cassey et al., 2008).

Functional composition also differed significantly across study conditions and city regions. While some contrasts were not statistically distinguishable, these non‐significant comparisons showed no clear clustering, nor did they suggest convergence among urban assemblages. Rather than indicating homogenisation, these patterns point to a context‐dependent filtering process in which urbanisation selects different subsets of the regional species pool across cities, as suggested in the past (Sol et al., 2014; La Sorte et al., 2018). Notably, although the aforementioned few widespread, urban‐associated species occurred consistently across urban sites, this limited overlap did not translate into convergence in either incidence‐based or abundance‐weighted functional composition.

Among the non‐significant contrasts, some may reflect context‐dependent functional convergence, where different environmental drivers, such as aridity and urbanisation, lead to assemblages with similar functional attributes despite differences in species composition. However, these findings should be interpreted within the broader context of generally high dissimilarity values among conditions and cities and without a consistent pattern of clustering among urban sites. Incidence‐based functional analyses were consistent with taxonomic composition patterns. Conversely, abundance‐based functional composition was primarily structured by local context and regional species pools, rather than by a process of urban‐driven homogenisation. In particular, Morelia's urban sites stood out due to the dominance of House Sparrows and Rock Pigeons, which aligns with the fact that the studied functional traits are associated with resource use and generalist strategies (Kark et al., 2007; Neate‐Clegg et al., 2023). Other conditions across cities showed no consistent clustering, reinforcing the absence of a clear homogenisation signal.

## 5 CONCLUSION

Taken together, our results show that assumptions of urban‐driven biotic and functional homogenisation are not generalisable, highlighting the need to reassess this paradigm in urban ecology, moving from assumed homogenisation toward empirically quantified, context‐dependent community responses. While urbanisation filters species and simplifies communities in terms of richness, the studied urban bird communities retained regional structure without clear convergence into homogenised assemblages, with both taxonomic and functional turnover shaped by context‐dependent filters acting on local species pools and environmental conditions. Widespread urban‐associated species, although sometimes highly abundant, contributed little to these patterns and did not appear to drive convergence across cities, even when abundance‐weighted metrics were considered. These results underscore the importance of empirically quantifying community responses, carefully considering methodological choices and reconsidering long‐standing assumptions about urban‐driven biotic and functional homogenisation in ecological studies.

## AUTHOR CONTRIBUTIONS

IM‐F and JES conceived the study. IM‐F conducted the fieldwork. IM‐F and BFL performed the data analyses, and IM‐F and CCR‐B interpreted the results. All authors contributed to the writing of initial drafts, and to the reviewing and editing the manuscript and approved the final version.

## CONFLICT OF INTEREST STATEMENT

The authors confirm that no conflicts of interest exist and that this has been stated in the manuscript.

## ETHICS STATEMENT

This study did not require ethical approval, as all data were obtained through observational sampling in publicly accessible sites without any direct interaction with humans or handling of vertebrate animals.

## Supporting information


**Table S1.** Recorded bird species and their abundance across the studied conditions per city region at Hermosillo, Morelia and Tuxtla Gutiérrez, along with their functional group as defined by the method used in this study.
**Table S2.** Recorded bird species and their dominant diet, mass (g), and foraging strata retrieved from Wilman et al. (2014).

## Data Availability

Data supporting the findings can be found in Zenodo https://doi.org/10.5281/zenodo.20959651.
