# Supplementary material for: Filtered, yet not homogenised: Urbanisation does not lead to cross‐city convergence in Mexican urban bird communities
Source: J Anim Ecol. 2026 Sep 1;95(10):1933–43. doi: 10.1111/1365-2656.70311 (PMC13643177; doi:10.1111/1365-2656.70311)
Supplement: Supplementary file 1 [file JANE-95-1933-s001.docx]

Table S1. Recorded bird species and their abundance across the studied conditions per city region at Hermosillo, Morelia, and Tuxtla Gutiérrez, along with their functional group as defined by the method used in this study. Taxonomy follows that of the AOU (2024), and the display order is based on functional group. Nat. = Natural ecosystem. Green. = Greenspaces

Species	Common name	Hermosillo	Morelia	Tuxtla Gutiérrez

Nat.	Green.	Urban	Nat.	Green.	Urban	Nat.	Green.	Urban

*Euphonia hirundinacea*	Yellow-throated Euphonia	0	0	0	0	0	0	1	1	0

*Myadestes occidentalis*	Brown-backed Solitaire	0	0	0	19	0	0	0	0	0

*Phainopepla nitens*	Phainopepla	5	0	0	0	0	0	0	0	0

*Eupsittula canicularis*	Orange-fronted Parakeet	0	0	0	0	0	0	0	5	1

*Aulacorhynchus prasinus*	Northern Emerald-Toucanet	0	0	0	0	0	0	1	0	0

*Ortalis vetula*	Plain Chachalaca	0	0	0	0	0	0	6	0	0

*Peucaea carpalis*	Rufous-winged Sparrow	1	0	0	0	0	0	0	0	0

*Amaurospiza concolor*	Blue Seedeater	0	0	0	0	0	0	1	0	0

*Spinus pinus*	Pine Siskin	0	0	0	1	0	0	0	0	0

*Spinus psaltria*	Lesser Goldfinch	0	0	0	4	22	2	0	8	5

*Haemorhous mexicanus*	House Finch	3	7	6	0	9	0	0	0	1

*Passer domesticus*	House Sparrow	0	26	27	0	31	75	0	1	17

*Sporophila torqueola*	Cinnamon-rumped Seedeater	0	0	0	0	14	4	0	2	1

*Volatinia jacarina*	Blue-black Grassquit	0	0	0	0	0	0	0	2	0

*Agelaius phoeniceus*	Red-winged Blackbird	1	0	0	0	0	0	0	0	0

*Columbina inca*	Inca Dove	8	13	11	2	20	14	0	17	15

*Molothrus aeneus*	Bronzed Cowbird	2	0	1	0	1	2	0	5	0

*Melozone fusca*	Canyon Towhee	1	0	0	3	33	11	0	0	0

*Melanerpes formicivorus*	Acorn Woodpecker	0	0	0	8	0	0	0	0	0

*Zenaida macroura*	Mourning Dove	18	29	21	0	0	0	0	0	0

*Leptotila verreauxi*	White-tipped Dove	0	0	0	5	0	0	11	0	0

*Zenaida asiatica*	White-winged Dove	9	24	10	0	0	0	0	0	0

*Columba livia*	Rock Pigeon	0	34	10	0	4	17	0	0	9

*Auriparus flaviceps*	Verdin	3	0	1	0	0	0	0	0	0

*Basileuterus rufifrons*	Rufous-capped Warbler	0	0	0	9	0	0	2	0	0

*Camptostoma imberbe*	Northern Beardless-Tyrannulet	0	0	3	0	0	0	0	0	0

*Catherpes mexicanus*	Canyon Wren	0	0	0	0	2	16	0	0	0

*Chaetura vauxi*	Vaux's Swift	0	0	0	0	0	2	0	1	0

*Empidonax oberholseri**	Dusky Flycatcher	0	0	0	7	0	0	0	0	0

*Empidonax difficilis*	Western Flycatcher	0	0	0	6	0	0	0	0	0

*Cardellina rubra*	Red Warbler	0	0	0	1	0	0	0	0	0

*Hirundo rustica*	Barn Swallow	0	0	0	0	14	27	0	0	0

*Myiarchus tuberculifer*	Dusky-capped Flycatcher	0	0	0	1	0	0	0	0	0

*Myioborus miniatus*	Slate-throated Redstart	0	0	0	23	0	0	0	0	0

*Oreothlypis superciliosa*	Crescent-chested Warbler	0	0	0	4	0	0	0	0	0

*Peucedramus taeniatus*	Olive Warbler	0	0	1	1	0	0	0	0	0

*Polioptila albiloris*	White-lored Gnatcatcher	0	0	0	0	0	0	1	0	0

*Polioptila melanura*	Black-tailed Gnatcatcher	6	0	0	0	0	0	0	0	0

*Pyrocephalus rubinus*	Vermilion Flycatcher	0	0	0	0	7	1	0	0	0

*Salpinctes obsoletus*	Rock Wren	6	0	0	0	0	0	0	0	0

*Tachycineta thalassina*	Violet-green Swallow	3	0	0	0	0	0	0	0	0

*Thryomanes bewickii*	Bewick's Wren	0	0	0	9	7	0	0	0	0

*Cantorchilus modestus*	Cabanis's Wren	0	0	0	0	0	0	0	1	0

*Thryophilus pleurostictus*	Banded Wren	0	0	0	0	0	0	10	0	0

*Troglodytes aedon*	Northern House Wren	0	0	0	4	5	0	0	0	0

*Vireo bellii*	Bell's Vireo	1	0	0	0	0	0	0	0	0

*Aeronautes saxatalis*	White-throated Swift	0	0	0	0	0	1	0	0	1

*Campylorhynchus brunneicapillus*	Cactus Wren	21	0	0	0	0	0	0	0	0

*Campylorhynchus gularis*	Spotted Wren	0	0	0	3	0	0	0	0	0

*Catharus aurantiirostris*	Orange-billed Nightingale-Thrush	0	0	0	0	0	0	1	0	0

*Catharus occidentalis*	Russet Nightingale-Thrush	0	0	0	2	0	0	0	0	0

*Contopus pertinax*	Greater Pewee	0	0	0	14	0	0	0	0	0

*Habia rubica*	Red-crowned Ant-Tanager	0	0	0	0	0	0	1	0	0

*Icterus bullockii*	Bullock's Oriole	0	0	0	1	0	0	0	0	0

*Icterus galbula*	Baltimore Oriole	0	0	0	0	0	0	0	2	0

*Icterus pustulatus*	Streak-backed Oriole	0	0	0	0	0	0	6	7	1

*Icterus wagleri*	Black-vented Oriole	0	0	0	0	0	0	1	0	0

*Lepidocolaptes leucogaster*	White-striped Woodcreeper	0	0	0	6	0	0	0	0	0

*Molothrus ater*	Brown-headed Cowbird	2	0	0	0	0	0	0	0	0

*Myiarchus cinerascens*	Ash-throated Flycatcher	1	0	0	0	0	0	0	0	0

*Myiozetetes similis*	Social Flycatcher	0	0	0	0	0	0	0	4	1

*Passerina caerulea*	Blue Grosbeak	0	0	0	0	2	1	0	0	0

*Dryobates scalaris*	Ladder-backed Woodpecker	0	0	0	0	1	0	0	0	0

*Piranga flava*	Hepatic Tanager	0	0	0	11	1	0	0	0	0

*Sialia sialis*	Eastern Bluebird	0	0	0	4	0	0	0	0	0

*Tyrannus melancholicus*	Tropical Kingbird	1	1	0	0	0	0	0	4	12

*Tyrannus verticalis*	Western Kingbird	12	6	4	0	0	0	0	0	0

*Chordeiles acutipennis*	Lesser Nighthawk	1	0	0	0	0	0	0	0	0

*Mimus gilvus*	Tropical Mockingbird	0	0	0	0	0	0	0	2	0

*Tyrannus crassirostris*	Thick-billed Kingbird	0	2	0	0	0	0	0	0	0

*Tyrannus vociferans*	Cassin's Kingbird	0	0	0	0	2	1	0	0	0

*Cassiculus melanicterus*	Yellow-winged Cacique	0	0	0	0	0	0	5	2	0

*Crotophaga sulcirostris*	Groove-billed Ani	0	0	0	0	1	0	5	6	0

*Glaucidium brasilianum*	Ferruginous Pygmy-Owl	0	0	0	0	0	0	0	1	0

*Megarynchus pitangua*	Boat-billed Flycatcher	0	0	0	0	0	0	0	1	0

*Momotus mexicanus*	Russet-crowned Motmot	0	0	0	0	0	0	6	1	0

*Streptoprocne zonaris*	White-collared Swift	0	0	0	0	0	0	0	0	1

*Toxostoma curvirostre*	Curve-billed Thrasher	6	0	0	0	4	0	0	0	0

*Turdus migratorius*	American Robin	0	0	0	1	1	0	0	0	0

*Turdus rufopalliatus*	Rufous-backed Robin	0	0	0	0	17	1	0	0	0

*Colaptes chrysoides*	Gilded Flicker	3	0	0	0	0	0	0	0	0

*Piaya cayana*	Squirrel Cuckoo	0	0	0	0	0	0	1	0	0

*Dryocopus lineatus*	Lineated Woodpecker	0	0	0	0	0	0	1	0	0

*Geococcyx californianus*	Greater Roadrunner	2	0	0	0	0	0	0	0	0

*Saucerottia beryllina*	Berylline Hummingbird	0	0	0	10	2	0	3	9	1

*Cynanthus latirostris*	Broad-billed Hummingbird	1	3	4	1	11	6	0	0	0

*Basilinna leucotis*	White-eared Hummingbird	0	0	0	3	0	0	0	0	0

*Eugenes fulgens*	Rivoli's Hummingbird	0	0	0	1	0	0	0	0	0

*Heliomaster constantii*	Plain-capped Starthroat	0	0	0	0	0	0	1	0	0

*Amphispiza bilineata*	Black-throated Sparrow	1	0	0	0	0	0	0	0	0

*Arremonops rufivirgatus*	Olive Sparrow	0	0	0	0	0	0	3	0	0

*Atlapetes pileatus*	Rufous-capped Brushfinch	0	0	0	3	0	0	0	0	0

*Icterus cucullatus*	Hooded Oriole	0	1	1	0	0	0	0	0	0

*Junco phaeonotus*	Yellow-eyed Junco	0	0	0	1	0	0	0	0	0

*Spizella atrogularis*	Black-chinned Sparrow	0	0	0	5	0	0	0	0	0

*Spizella passerina*	Chipping Sparrow	0	0	0	3	0	0	0	0	0

*Cardinalis sinuatus*	Pyrrhuloxia	7	0	0	0	0	0	0	0	0

*Columbina passerina*	Common Ground Dove	8	0	0	1	0	0	1	0	0

*Mimus polyglottos*	Northern Mockingbird	6	0	1	0	0	0	0	0	0

*Myiodynastes luteiventris*	Sulphur-bellied Flycatcher	0	0	0	1	0	0	2	0	0

*Pipilo erythrophthalmus*	Eastern Towhee	0	0	0	11	0	0	0	0	0

*Ptiliogonys cinereus*	Gray Silky-flycatcher	0	0	0	0	4	0	0	0	0

*Cyanocorax yncas*	Green Jay	0	0	0	0	0	0	4	0	0

*Dives dives*	Melodious Blackbird	0	0	0	0	0	0	1	0	0

*Melanerpes aurifrons*	Golden-fronted Woodpecker	0	0	0	0	28	2	9	25	4

*Melanerpes uropygialis*	Gila Woodpecker	10	33	7	0	0	0	0	0	0

*Melanotis caerulescens*	Blue Mockingbird	0	0	0	3	0	0	0	0	0

*Pipilo ocai*	Collared Towhee	0	0	0	2	0	0	0	0	0

*Pitangus sulphuratus*	Great Kiskadee	0	0	0	0	0	0	3	10	3

*Toxostoma bendirei*	Bendire's Thrasher	1	0	0	0	0	0	0	0	0

*Trogon elegans*	Elegant Trogon	0	0	0	1	0	0	0	0	0

*Trogon melanocephalus*	Black-headed Trogon	0	0	0	0	0	0	2	0	0

*Turdus grayi*	Clay-colored Thrush	0	0	0	0	0	0	18	20	4

*Aphelocoma ultramarina*	Transvolcanic Jay	0	0	0	3	0	0	0	0	0

*Cyanocitta stelleri*	Steller's Jay	0	0	0	2	0	0	0	0	0

*Falco sparverius*	American Kestrel	3	2	0	0	0	0	0	0	0

*Callipepla gambelii*	Gambel's Quail	2	0	0	0	0	0	0	0	0

*Quiscalus mexicanus*	Great-tailed Grackle	0	11	16	0	8	8	0	28	36

*Streptopelia decaocto*	Eurasian Collared-Dove	0	0	1	0	0	0	0	0	0

*Amazona albifrons*	White-fronted Parrot	0	0	0	0	0	0	0	3	0

*Cyanocorax formosus*	White-throated Magpie-Jay	0	0	0	0	0	0	1	0	0

*Ardea ibis*	Western Cattle-Egret	0	0	0	0	1	0	0	0	0

*Corvus corax*	Common Raven	1	0	0	1	0	0	0	0	0

*Cathartes aura*	Turkey Vulture	1	0	1	0	0	0	0	0	0

*Coragyps atratus*	Black Vulture	0	0	0	0	0	0	3	0	0

*Accipiter striatus*	Sharp-shinned Hawk	0	0	0	0	1	0	0	0	0

*Egretta thula*	Snowy Egret	0	0	0	0	0	0	1	0	0

*Ardea alba*	Great Egret	0	1	0	0	0	0	0	0	0

*Buteo jamaicensis*	Red-tailed Hawk	1	0	0	0	0	0	0	0	0

*Buteo swainsoni*	Swainson's Hawk	2	0	0	0	0	0	0	0	0

*Eudocimus albus*	White Ibis	0	0	1	0	0	0	0	0	0

*Combined with records of *E. hammondii* (Hammond’s Flycatcher) as they are nearly indistinguishable in the field.

Table S2. Recorded bird species and their dominant diet, mass (g), and foraging strata retrieved from Wilman et al. (2014). Taxonomy follows that of the AOU (2024), and the display order is alphabetical.

Species	Dominant diet	Mass	Foraging strata*

*Accipiter striatus*	VertFishScav	130.59	3.5

*Aeronautes saxatalis*	Invertebrate	32.1	4.6

*Agelaius phoeniceus*	Omnivore	50.78	1.1

*Amaurospiza concolor*	PlantSeed	13.1	1.8

*Amazona albifrons*	PlantSeed	206	2.5

*Amphispiza bilineata*	Omnivore	13.5	1

*Aphelocoma ultramarina*	Omnivore	113.33	1.7

*Ardea alba*	Omnivore	85	2.5

*Ardea ibis*	Invertebrate	365.95	0.7

*Arremonops rufivirgatus*	Omnivore	22.43	1.2

*Atlapetes pileatus*	Omnivore	24	1.3

*Aulacorhynchus prasinus*	Omnivore	170.56	3.4

*Auriparus flaviceps*	VertFishScav	871.33	0.5

*Basileuterus rufifrons*	Invertebrate	10.4	2

*Basilinna leucotis*	FruiNect	3.39	2.7

*Buteo jamaicensis*	VertFishScav	1101.16	3.96

*Buteo swainsoni*	VertFishScav	946.61	3

*Callipepla gambelii*	PlantSeed	165.95	1

*Camptostoma imberbe*	Invertebrate	7.4	3.3

*Campylorhynchus brunneicapillus*	Invertebrate	38.9	1.5

*Campylorhynchus gularis*	Invertebrate	30.1	1.98

*Cantorchilus modestus*	Invertebrate	17.57	2.6

*Cardellina rubra*	Invertebrate	8.1	2.3

*Cardinalis sinuatus*	PlantSeed	35.19	1.7

*Cassiculus melanicterus*	Invertebrate	81.34	3.2

*Cathartes aura*	VertFishScav	1518.24	1

*Catharus aurantiirostris*	Omnivore	29.8	1.2

*Catharus occidentalis*	Omnivore	26.2	2

*Catherpes mexicanus*	Invertebrate	11.97	1

*Chaetura vauxi*	Invertebrate	17.1	5

*Chordeiles acutipennis*	Invertebrate	48.42	3

*Colaptes chrysoides*	Invertebrate	111	1.6

*Columba livia*	PlantSeed	354.2	1.2

*Columbina inca*	PlantSeed	47.5	1

*Columbina passerina*	Omnivore	35.36	1

*Contopus pertinax*	Invertebrate	27.2	3

*Coragyps atratus*	VertFishScav	1881.69	1

*Corvus corax*	VertFishScav	927.97	1.6

*Crotophaga sulcirostris*	Invertebrate	82.04	1.1

*Cyanocitta stelleri*	Omnivore	128	2.2

*Cyanocorax formosus*	Omnivore	210	2.2

*Cyanocorax yncas*	Omnivore	78.5	2.3

*Cynanthus latirostris*	FruiNect	3.1	2.5

*Dives dives*	Invertebrate	91.32	1.3

*Dryobates scalaris*	Invertebrate	31.59	2.8

*Dryocopus lineatus*	Invertebrate	183.19	2.4

*Egretta thula*	Omnivore	371	0.3

*Empidonax difficilis*	Invertebrate	11.6	2.1

*Empidonax oberholseri*	Invertebrate	10.44	1.98

*Eudocimus albus*	VertFishScav	889.66	0.6

*Eugenes fulgens*	FruiNect	8	2.6

*Euphonia hirundinacea*	FruiNect	14	3.3

*Eupsittula canicularis*	Invertebrate	6.8	1.3

*Falco sparverius*	Invertebrate	114.61	1.6

*Geococcyx californianus*	VertFishScav	376	1

*Glaucidium brasilianum*	Invertebrate	75.1	1.3

*Habia rubica*	Invertebrate	32.46	2.6

*Haemorhous mexicanus*	PlantSeed	21.4	1.98

*Heliomaster constantii*	FruiNect	7.3	3.1

*Hirundo rustica*	Invertebrate	17.91	2.1

*Icterus bullockii*	Invertebrate	37.9	2.5

*Icterus cucullatus*	Omnivore	24.3	2.5

*Icterus galbula*	Invertebrate	32.83	2.7

*Icterus pustulatus*	Invertebrate	36.78	3.2

*Icterus wagleri*	FruiNect	41.8	3.1

*Junco phaeonotus*	PlantSeed	20.4	1.2

*Lepidocolaptes leucogaster*	Invertebrate	36	2.5

*Leptotila verreauxi*	PlantSeed	146.88	1

*Megarynchus pitangua*	Invertebrate	69.91	3.3

*Melanerpes aurifrons*	Omnivore	80.77	2.4

*Melanerpes formicivorus*	PlantSeed	79.62	3.6

*Melanerpes uropygialis*	Omnivore	64.66	2.6

*Melanotis caerulescens*	Invertebrate	61.57	1.5

*Melozone fusca*	PlantSeed	44.4	1.7

*Mimus gilvus*	Invertebrate	51.99	1.5

*Mimus polyglottos*	Omnivore	48.5	1.3

*Molothrus aeneus*	Invertebrate	62.61	1.3

*Molothrus ater*	PlantSeed	40.26	1.3

*Momotus mexicanus*	Invertebrate	75.7	1.8

*Myadestes occidentalis*	Invertebrate	36.4	2.8

*Myiarchus cinerascens*	Invertebrate	28.2	2.3

*Myiarchus tuberculifer*	Invertebrate	17.7	2.97

*Myioborus miniatus*	Invertebrate	9	3

*Myiodynastes luteiventris*	Omnivore	46.9	4.5

*Myiozetetes similis*	Omnivore	28	2.6

*Oreothlypis superciliosa*	Invertebrate	9	3

*Ortalis vetula*	FruiNect	562.6	3.5

*Passer domesticus*	PlantSeed	26.51	1.5

*Passerina caerulea*	Omnivore	27.39	1

*Peucaea carpalis*	PlantSeed	15.3	1.6

*Peucedramus taeniatus*	Invertebrate	11	2

*Phainopepla nitens*	FruiNect	22.1	3

*Piaya cayana*	Invertebrate	101.98	3.8

*Pipilo erythrophthalmus*	Omnivore	40.03	1.2

*Pipilo ocai*	Omnivore	60.95	1.4

*Piranga flava*	Invertebrate	37.7	3.5

*Pitangus sulphuratus*	Omnivore	62.85	1.6

*Polioptila albiloris*	Invertebrate	6.26	2.5

*Polioptila melanura*	Invertebrate	5.2	2.5

*Ptiliogonys cinereus*	Omnivore	33.6	2.5

*Pyrocephalus rubinus*	Invertebrate	14.4	2.5

*Quiscalus mexicanus*	Invertebrate	160.47	1.3

*Salpinctes obsoletus*	Invertebrate	16.37	1

*Saucerottia beryllina*	FruiNect	4.5	3

*Sialia sialis*	Invertebrate	27.5	1

*Spinus pinus*	PlantSeed	12.7	2

*Spinus psaltria*	PlantSeed	8.77	2.2

*Spizella atrogularis*	Omnivore	11.3	1

*Spizella passerina*	PlantSeed	12.2	1.3

*Sporophila torqueola*	PlantSeed	8.7	1.8

*Streptopelia decaocto*	PlantSeed	148.96	1.3

*Streptoprocne zonaris*	Invertebrate	86.79	5

*Tachycineta thalassina*	Invertebrate	14.14	3

*Thryomanes bewickii*	Invertebrate	9.9	1.5

*Thryophilus pleurostictus*	Invertebrate	17.7	1.8

Toxostoma bendirei	Omnivore	62.19	1

Toxostoma curvirostre	Omnivore	80.54	1

*Troglodytes aedon*	Invertebrate	10.85	2

*Trogon elegans*	Omnivore	70.89	3.7

*Trogon melanocephalus*	Omnivore	85.1	2.9

*Turdus grayi*	FruiNect	79.5	2.8

*Turdus migratorius*	Omnivore	78.5	2.2

*Turdus rufopalliatus*	Invertebrate	75.02	1

*Tyrannus crassirostris*	Invertebrate	55.89	3.9

*Tyrannus melancholicus*	Invertebrate	37.4	3.5

*Tyrannus verticalis*	Invertebrate	39.6	2.1

*Tyrannus vociferans*	Invertebrate	45.6	2.8

*Vireo bellii*	Invertebrate	8.5	2.5

*Volatinia jacarina*	Omnivore	9.94	1

*Zenaida asiatica*	PlantSeed	153	1.5

*Zenaida macroura*	PlantSeed	118.93	1.4

*Assigned numeric values reflecting vertical foraging position (0 = ground, 1 = understory, 2 = mid-high, 3 = canopy, and 4 = aerial) calculated using a weighted value from the percentages in the dataset, producing a single numerical value for each species, ranging from 0 to 4, where 0 corresponds to exclusively ground foraging and 4 to exclusively aerial foraging.
